# Remarks on the Prevention of Scrivener’s Palsy or Writer’s Cramp

**Published:** 1886-12

**Authors:** Frank Woodbury

**Affiliations:** Professor of Therapeutics, Materia Medica, and Clinical Medicine in the Medico-Chirurgical College, Philadelphia


### COMMUNICATIONS.

### Remarks on the Prevention of Scrivener's Palsy or Writer's Cramp.

(Read before the Philadelphia County Medical Society at a meeting held September 8th, 1886.)

BY FRANK WOODBURY, M.D.,

Professor of Therapeutics, Materia Medica, and Clinical Medicine in the Medico-Chirurgical College, Philadelphia.

Among the compensations required by the high state of civilization in which we live are a class of disorders to which Benedict has applied the rather unwieldy title of "Co-ordinated Business Neuroses" (Co-ordinatorische Beschaftungsneurosen), and which are usually grouped under the name of Professional Dyskinesia, or Anapeiretic Paralysis (Hammond). They are met with in every walk of life; clerks, stenographers, telegraph operators, type-setters, tailors, carpenters, watchmakers, blacksmiths, musicians, milk-maids, seamstresses, ballet-dancers, and in short, all who are called upon to perform delicate, more or less complex, muscular movements, which are repeated over and over again, with unvarying regularity, day by day, month by month, year after year. As those persons who use the pen constantly in writing are by far the most numerous of these professional laborers, it is not surprising that cases of writer's cramp or scrivener's palsy are frequently encountered, or that this condition has been studied more thoroughly than the other forms of professional paralysis. In fact, it is so well known to all that a description of the affection is rendered unnecessary in the way of introduction to the few remarks which I have to offer upon some of the means that may be adopted for its prevention.

With regard to the question of diagnosis, however, I might be permitted to observe that Graphospasm can be readily distinguished, by the exercise of ordinary care, from disorder of the writing faculty accompanying hemiplegia (Agraphia), locomotor ataxia, and progressive muscular atrophy (the muscular disease), or that due to neuritis of the branches of the brachial plexus, or produced by pressure upon the nerve trunks by a tumor. Dr. Poore, for instance, reports a case of progressive difficulty in writing, going on to complete illegibility, which was caused by the pressure of a subclavio-axillary aneurism. Of course, no one need confound writer's cramp with brachial monoplegia, lead-palsy, or rheumatic paralysis of the fore-arm, although such mistakes have been made.

The characters peculiar to artisan's cramp are weakness and spasm. These are not always experienced, however, as the attacks may come on, without premonition, immediately upon attempting to do certain work, or after it has been performed with comparative facility for a greater or less period of time. All writers upon the subject recognize a strong emotional element in the disease. McLane Hamilton, who regards the disorder as due to excessively developed automatism, claims that those who write and compose at the same time are less likely to acquire the disease than clerks and copyists who do "machine work." My own observation leads me to believe with Poore, that the contrary is the case. That is to say, the amount of actual work performed being the same, the person whose mind is most exercised will be more likely to be attacked by writer's cramp than he who merely writes automatically. This view is sustained by experiment as well as by observation. Drs. Dercum and Parker found from a series of experiments that convulsions could be produced by subjecting a group of muscles to constant and precise effort, the attention being at the same time concentrated upon some train of thought.*

The problem of prevention, fortunately, is not materially affected by the very different views which are held as to the pathology. Erb regards it as essentially a nerve disorder, involving the central ganglia which affect the association and co-ordination of the muscular movements for the more complex actions. Dr. Frank Smith discovered in certain cases of ham-

* Quoted from Dr. Lewis's article on the Nervous Disorders of Writers and Artisans. Pepper's System of Medicine, Philadelphia, 1886, Vol. 5, p. 529.

merman's palsy, (hephæstic paralysis) lesions in the cerebral hemispheres. Reynolds, Ross, and Hammond regard writer's spasm in like manner to be due to disorder of the spinal motor cells and cerebral nerve motor centres, although our present means of investigation may not permit us in each case to detect the precise anatomical lesion. The other view is that of Zuradelli, ably defended by Dr. Poore, that the starting point of the morbid change is the exhaustion of certain intrinsic muscles of the hand which are most taxed in writing. "One or more of these begins after a time to respond sluggishly, or not at all, to the stimulus of the will. The patient then unconsciously calls into play other muscles, generally those of the fore-arm. In their turn, these too become worn out, and the process of substitution is carried on indefinitely, and always with the same result."* It is possible, even highly probable, that both nerve centre and muscle suffer in their nutrition in well-marked cases of writer's cramp.

Two forms have been distinguished, depending upon whether the spasm or the paresis is the more prominent feature in the individual case, and this distinction which has no great value, is shown in the popular names of the disorder, writer's cramp and scrivener's palsy.

Although relief may be afforded by galvanism, massage, and passive movements, as in Wolff's method, the affection is scarcely susceptible of complete cure. For this reason the prevention of such a disabling malady becomes of the first importance.

Before discussing the means of prevention, it should be stated that a hereditary element must be recognized in this as in other functional disorders, and descendants of persons subject to any form of artisan's cramp must adopt extraordinary precautions if they would escape from being attacked themselves.

The first of the means to which I invite attention is the manner of holding the pen in writing. I wish to insist upon the correctness of the view which regards a faulty system of writing as the most prolific cause of this disorder. I maintain that while the

* Fagge's Practice of Medicine, Philadelphia, 1886, Vol. 1, p. 641.

pen is *held* by the fingers, the movements of writing should be made by the hand and not by the fingers. Children at school should never be allowed to write or work their exercises with short stumps of pencils. Whenever the hand is engaged in the act of writing, the pencil or pen-holder should be long enough to pass from the tip of the second finger to a point a little beyond the metacarpo-phalangeal joint of the first finger, being held in this position by a light pressure of the thumb, the fore-finger lightly resting upon the upper surface of the holder just above the pen. The anterior surface of the fore-arm is allowed to rest upon the the desk or table, while the whole hand, supported upon the extremity of the little finger, moves with each stroke of the pen. Writing by this method is less fatiguing than when done principally by the fingers. Writing with steel pens has been accused of causing the affection under consideration, but the resort to a " good gray goose-quill" is certainly no safeguard against it. It is a fact, however, that those who have much of this kind of work to do usually prefer a gold pen.

The character of the pen-holder is by no means unimportant, especially in the early stage of the disorder. Some authorities recommend, instead of the pen, to use a lead-pencil only or the stylograph or fluid pencil. Many pen-holders have a smooth metal barrel or sheath for the pen, which, especially in warm weather, has a disagreeable slippery or greasy feeling to the fingers. Although not much importance is to be attached to the statement that electrical currents are generated by the scratching of the pen upon the surface of the paper, it will be found that decided relief is obtained from having the holder made of some non-conducting material, such as hard rubber or cork. A simple but very useful device I show here. It is a soft rubber tube or shield, which is to be slipped over the part of the pen-holder which comes in contact with the fingers. The amount of comfort to be obtained from this by persons who are very sensitive to peripheral impressions is entirely out of proportion to its trifling cost.

In cases where the spasm is a marked feature, and the patient finds himself stabbing the paper with his pen, I think that a form of holder which I invented some years ago might be serviceable by keeping the point of the pen at a uniform degree of pressure upon the paper, and by imparting a sense of security to the patient. This instrument was mentioned by Dr. D. F. Lincoln in his work on "School and Industrial Hygiene," published by Blakiston in 1880, but it has not previously been shown before any society.*

The only peculiarity about this pen-holder for the prevention of writer's cramp is the separation of the pen and its holder from the portion which is grasped by the fingers in the act of writing. The instrument is an ordinary pen-holder with the addition of an outside sleeve surrounding its lower portion, which is held in place by a concealed spring, and which is movable in one direction (downward) to the extent of about half an inch. The object of this is that if undue prussure be made upon the point of the pen, the spring will yield in proportion to the force and allow the fingers to be extended without perforating the paper; the usual relative position of the fingers and pen will be at once restored as soon as the pressure is intermitted. This pen-holder could be made in several sizes to suit different persons, since some prefer a thick holder; and the stick could be made of cork or hollow vulcanized rubber so as to secure the necessary lightness.

Variously shaped pen-holders have been constructed for the purpose of assisting the subjects of this annoying affection to write. One of Dr. Poor's patients used a pen-holder in the shape of a "top," which he grasped with his whole hand. Velpeau's well-known apparatus consists of an oblong ball, made of wood or hard rubber, to be held in the hand. To this are attached a pair of half-rings, which serve as rests for the index and middle fingers. A pen-holder passes through the neck of the ball at a convenient place near the extremity, which can be adjusted and made firm by a thumb-screw. With this apparatus a person affected with writer's cramp may be enabled to do a certain amount of writing that under ordinary circumstaces would be entirely beyond his powers to accomplish. Matthieu's instrument is also supplied by the surgical instrument makers. It consists of two rings for the index finger, and a half-ring properly distanced from the others, on which to rest the thumb. To these is attached a spring slide, into which a common pen-holder can be inserted. These devices, however, are useful where the disorder already exists, and are never employed with a view to its prevention.

Until the phonograph, or some modification of it shall come into general use, a large proportion of the permanent record of the daily work of the world must be committed to writing by hand. This brings up the very interesting question of a substitute for the pen. One of the most useful of modern inventions is the type-writer, which I consider very efficient in preventing writer's cramp. It is now largely in use among business men, but especially is it being adopted by authors, ministers, lawyers, editors; all of whom are accustomed to compose as they write, and are therefore, in my opinion, more liable to be attacked by this annoying disability. Dr. Lincoln, who is Chairman of the Department of Health of the Social Science Association, considers it serviceable even after the disorder has been developed: in the work from which I have already quoted, he says, "the temporary use of the type-writing machine will often prove a great boon, permitting a continuation of work while resting the affected muscles."

In comparison with the ordinary method of writing, the advantage is all on the side of the type-writer. Instead of having one hand actively employed in performing very rapid and highly complicated movements while the other is almost absolutely idle (and probably this equally applies to the motor areas of the cerebral hemispheres), we see both hands engaged in movements, which suggest playing on the piano, but are less laborious. From personal experience I can say that with its aid, copying or extemporaneous writing is less fatiguing than with the pen.

A recent editorial in the Journal of the American Medical Association* entitled "Medical Writers and Type-writers:" concludes with a paragraph which is so appropriate that I will quote it in bringing this article to a close. The editor says:

---

* Journal of the American Medical Association, July 17, p. 22, Vol. vii., No. 3.

"There are several reasons why medical men who write should use the type-writer. In the first place as a matter of economy of time, and hence, often of money. It is easily seen that there is a great saving of time with a machine that will do a piece of work in one-half or one-third the time that it can be done by hand; and this is easily accomplished with a type-writer. In the second place, the machine promotes accuracy, especially in thinking, for two reasons: being more rapid than the pen, the thoughts of the writer do not wander so far ahead of his work; and should he wish to refer to what has been written he can see it at a glance, as on a printed page. A third reason is, that the person who uses a type-writer sits erect, and can thus work for a longer time without fatigue than when bending over as in writing with a pen. It is thus more wholesome than the pen. The machine further promotes accuracy in the printing office—a great source of comfort to writers; and it promotes a degree of accuracy on the part oi the writer in that he will not make a wavy line, or two or three letters and an apostrophe, do duty for a long word, and his punctuation marks, if not more correct, will at least be more numerous.

"With a machine, therefore, writing becomes a pleasure, with none of the discomforts accidental and incidental to the use of the pen."

